# The natural history of primary progressive aphasia: beyond aphasia

**DOI:** 10.1007/s00415-021-10689-1

**Published:** 2021-07-03

**Authors:** Hulya Ulugut, Simone Stek, Lianne E. E. Wagemans, Roos J. Jutten, Maria Antoinette Keulen, Femke H. Bouwman, Niels D. Prins, Afina W. Lemstra, Welmoed Krudop, Charlotte E. Teunissen, Bart N. M. van Berckel, Rik Ossenkoppele, Frederik Barkhof, Wiesje M. van der Flier, Philip Scheltens, Yolande A. L. Pijnenburg

**Affiliations:** 1 Department of Neurology, Alzheimer Center Amsterdam, Amsterdam Neuroscience, Vrije Universiteit Amsterdam, Amsterdam UMC, De Boelelaan 1118, 1081 HZ Amsterdam, The Netherlands; 2 Department of Psychiatry, University Medical Center Utrecht, Utrecht, The Netherlands; 3 Neurological Laboratory Clinical Chemistry, Amsterdam Neuroscience, Vrije Universiteit Amsterdam, Amsterdam, The Netherlands; 4 Department of Radiology and Nuclear Medicine, Vrije Universiteit Amsterdam, Amsterdam UMC, Amsterdam, The Netherlands; 5 Clinical Memory Research Unit, Lund University, Lund, Sweden; 6 UCL Institutes of Neurology and Healthcare Engineering, University College London, London, UK; 7 Department of Epidemiology and Biostatistics, Amsterdam Neuroscience, Vrije Universiteit Amsterdam, Amsterdam UMC, Amsterdam, The Netherlands

**Keywords:** Dementia, Frontotemporal lobar degeneration, Frontotemporal dementia, Aphasia, Primary progressive aphasia, Mortality, Survival analysis, Natural history

## Abstract

**Introduction:**

Primary progressive aphasia (PPA) is divided into three prototypical subtypes that are all characterized by their single core symptom of aphasia. Although later in their course, other cognitive, behavioral, and motor domains may become involved, little is known about the progression profile of each subtype relative to the other subtypes.

**Methods:**

In this longitudinal retrospective cohort study, based on the recent biomarker-supported diagnostic criteria, 24 subjects diagnosed with semantic variant (svPPA), 22 with non-fluent variant (nfvPPA), and 18 with logopenic variant (lvPPA) were collected and followed up for 1–6 years. Symptom distribution, cognitive test and neuropsychiatric inventory scores, and progression into another syndrome were assessed.

**Results:**

Over time, lvPPA progressed with broader language problems (PPA-extended) and nfvPPA progressed to mutism, whereas semantic impairment remained the major problem in svPPA. Apart from linguistic problems, svPPA developed pronounced behavioral disturbances, whereas lvPPA exhibited a greater cognitive decline. By contrast, in nfvPPA motor deficits were more common. Furthermore, within 5 years (IQR = 2.5) after clinical onset, 65.6% of the patients additionally fulfilled the clinical criteria for another neurodegenerative syndrome (PPA-plus). Fourteen out of 24 (58%) svPPA patients additionally met the diagnostic criteria of behavioral variant frontotemporal dementia (5.1 years, IQR = 1.1), whereas the clinical features of 15/18 (83%) lvPPA patients were consistent with Alzheimer disease dementia (4.5 years IQR = 3.4). Furthermore, 12/22 (54%) of the subjects with the nfvPPA progressed to meet the diagnostic criteria of corticobasal syndrome, progressive supranuclear palsy, or motor neuron disease (5.1 years IQR = 3.4).

**Discussion:**

Despite aphasia being the initial and unique hallmark of the syndrome, our longitudinal results showed that PPA is not a language limited disorder and progression differs widely for each subtype, both with respect to the nature of symptoms and disease duration.

**Supplementary Information:**

The online version contains supplementary material available at 10.1007/s00415-021-10689-1.

## Introduction

Since the original description of the clinical syndrome of primary progressive aphasia (PPA) in six patients by Marsel Mesulam in 1982 [1], studies have focused on defining its clinical phenotypes, underlying molecular pathologies, and genetic background. Currently, the syndrome is divided into three variants: the semantic variant (svPPA), non-fluent/agrammatic variant (nfvPPA), and logopenic variant (lvPPA) [2]. SvPPA typically presents with loss of word and/or object meaning, decreased confrontation naming, and surface dyslexia associated with anterior temporal atrophy on neuroimaging. nfvPPA is characterized by effortful speech, reduced speech production, and agrammatism in the presence of impairment of the left posterior frontal and insular regions. The third syndromic variant is lvPPA presents with word-finding difficulties and repetition problems, while its radiological hallmark is temporo-parietal atrophy on the left side [2]. Although the previous literature has suggested that nfvPPA is the most heritable PPA variant (30–40% with a family history) [3], a study with a more detailed methodology showed that a clear autosomal dominant history is quite rare in all PPA subtypes [4]. On the other hand, while svPPA and nfvPPA are related with frontotemporal lobar degeneration (FTLD) pathologies, lvPPA links to Alzheimer’s disease pathology [5, 6].

It is known that the current diagnostic criteria do not cover all PPA patients and one-third to one-half of PPA syndromes is unclassifiable [7, 8]. Therefore, Mesulam and colleagues (2014) have used the term “PPA mixed” to designate the patients who have both comprehension deficits and grammatical errors, which is usually based on underlying Alzheimer’s disease pathology [9]. On the other hand, several other studies have shown that the unclassified group might present more complex language problems [7, 8, 10]. Another challenging issue for clinicians is that over the disease course, patients who initially perfectly fulfilled the diagnostic criteria for one of the PPA subtypes develop additional symptoms within and outside the language domain. Louwersheimer et al. (2015) have used the term “PPA extended” to cover those cases who fulfill the core criteria of one PPA subtype initially and subsequently progress with characteristic language symptoms of another PPA subtype [10], whereas Rogalski and Mesulam (2009) have proposed the term “PPA + (plus)” to specify the progression into other neurodegenerative syndromes accompanying the PPA diagnosis [11]. Nevertheless, to our knowledge, the patterns of the PPA-extended and PPA-plus forms of the three PPA subtypes have never been studied systematically in a well-categorized PPA cohort.

To date, the available longitudinal studies in PPA were either published before the publication of the current diagnostic criteria in 2011 or have focused on one subtype or one cognitive domain [12–16]. To our knowledge, two former longitudinal cohort studies have used the current classification and focused on the entire disease course of the syndrome [17, 18]. Unfortunately, the lack of information about the amyloid status of the subjects, as well as missing detailed descriptions of symptomatology make these studies difficult to interpret. In addition, an overall view on the progression profiles, and the patterns of PPA-extended and PPA-plus forms of the PPA subtypes are missing. This is crucial to provide adequate and satisfying information to patients about the prognosis of their disease as well as a roadmap to clinicians to better predict potential problems at the follow-up visits. Therefore, we set out to evaluate detailed symptom distribution, cognitive test performance, neuropsychiatric status, and progression into PPA-extended and PPA-plus, in each PPA subtype in a large memory clinic cohort.

## Methods

### Patient selection

One hundred twenty-six subjects who fulfilled the current diagnostic criteria of PPA [2] were included retrospectively from the Amsterdam Dementia Cohort [19] between January 2011 and March 2019. Since the aim of the study is to show the progression pattern of each subtype, the unclassified patients were excluded (*n* = 14). We also excluded the cases that at a closer look had the clinical profile and neuroimaging features of right temporal variant frontotemporal dementia (*n* = 5) [20]. This is important, because it has been shown that right temporal variant frontotemporal dementia is not a primary language disorder and it exhibits a different progression pattern compared to svPPA [20–22]. Of note, all excluded rtvFTD cases were right handed. Additionally, the cases that were not a Dutch native speaker (*n* = 3), had no records of amyloid status (*n* = 1), or had less than 1 year of clinical follow-up (*n* = 39) were also excluded. All remaining subjects had either CSF amyloid beta-42 levels (*n* = 54) or amyloid PET results (*n* = 32) available (Supplementary material 1). Their initial neuroimaging (MRI *n* = 62, CT n = 2, FDG-PET *n* = 14) met the radiological diagnostic criteria of PPA [2] (Supplementary Fig. 1). The eventual selection yielded a sample of 64 subjects with PPA, and based on the current diagnostic criteria [2], 24 subjects were diagnosed with svPPA, 22 with nfvPPA and 18 with lvPPA (Supplementary Fig. 2).

### Clinical assessment, longitudinal follow-up, and data collection

All subjects had undergone a detailed neurological and neuropsychological assessment at the initial visit, and all of them had been followed throughout their disease course by an experienced behavioral neurologist (Y.P or P.S). Family history of dementia was considered positive when the Modified Goldman score was 1 [4]. Education level was scored using the Verhage system [23].

The characteristic symptoms of dementia spectrum disorders were routinely recorded during the neurological interviews at our center. From the case notes, symptoms were clustered in the following groups; language/ speech, cognitive, behavioral/mood, and motor dysfunction (Supplementary material 2). Listed symptoms were recorded as present or absent for each subject for each visit and sub-classified as “initial symptoms” (at the initial visit) and “follow-up symptoms” (only rated when reported follow-up). After the initial visit, the subsequent visit in between 10 and 14 months was considered as “first year follow-up”, 22–26 months; “second year follow-up”, 34–38 months; “third year follow-up”, 46–50 months; “fourth year follow-up”, 58–62 months; “fifth year follow-up”, and 70–74 months; “sixth year follow-up”.

The following clinical data that are systematically recorded in our cohort were abstracted from all case notes: measures of functional severity [Clinical Dementia Rating Scale (CDR)], activities of daily living [Amsterdam instrumental activities of daily living (IADL) questionnaire], and the patients’ behavioral and psychological status [neuropsychiatric inventory (NPI)]. Cognitive functions were assessed with a standardized neuropsychological test battery, including global cognition [Mini Mental State Examination (MMSE)], episodic memory [visual association test (VAT) A and the Dutch version of the Rey Auditory Verbal Learning Test (RAVLT)], executive functions [Frontal assessment Battery (FAB) and digit span backward], semantic memory [category fluency animals], confrontation naming [VAT naming and Boston naming test], and visuospatial functions [Visual Objective and Space Perception (VOSP)-fragmented letters] [19].

The appearance of the progression into another PPA subtype was referred to as “PPA-extended [10] and the progression into another neurodegenerative syndrome was referred to as “PPA-plus” [11]. The time from aphasia onset to PPA-plus was based on the time up till meeting the diagnostic criteria for the second syndrome such as behavioral variant frontotemporal dementia (bvFTD), progressive supranuclear palsy (PSP), corticobasal syndrome (CBS), motor neuron disease (MND), and Alzheimer’s disease [24–28].

### Statistical analysis

Analyses were conducted using SPSS Statistics, version 24.0 (IBM) and R Studio (R Core Team, 2018).

Differences in frequencies of categorical variables between groups (svPPA, nfPPA, and lvPPA) were assessed with Chi-square and continuous variables were compared between groups with one-way ANOVA or Kruskal–Wallis analysis depending on the distribution of the variables based on Shapiro–Wilk normality test. Post hoc comparisons were corrected for multiple comparisons using the Bonferroni correction. Change over time in cognitive functioning was assessed using linear mixed models (LMM) with a random intercept and slope for each subject. Separate models were run for each cognitive test (dependent) with time (measured on a continuous level) as independent variable, separately for each diagnostic group. Nonparametric survival analyses were conducted using Kaplan–Meier estimates [inter-quartile range (IQR)] with post hoc Mantel Cox log rank tests to calculate progression into PPA-plus. The results were thresholded at a corrected *p* value of < 0.05.

### Standard protocol approvals, registrations, and patient consents

The local Medical Ethics Committee approved a general protocol for using the clinical data for research purposes (Protocol No: 2016.061).

### Data availability

Anonymized data can be made available by request to the corresponding author.

## Results

Table 1 displays the clinical and demographic features per diagnostic group. The gender distribution was almost equal in the lvPPA group. However, the majority of svPPA subjects were male, whereas the nfvPPA group was female predominant (*p* = 0.02). Mean age, mean symptom or follow-up duration, and the CDR and IADL scores did not differ between diagnostic groups. All svPPA patients were amyloid negative, whereas one (4%) nfvPPA and 15 (83%) lvPPA patients had a positive amyloid status (supplementary material 1). A few subjects were left-handed in all groups, but no statistical difference in the distribution of handedness was found (*p* = 0.86). Of note, to establish receptive language dominance in left-handed subjects, we checked whether clinical symptoms showed concordance with the anatomic distribution of cortical atrophy and clinical presentation. All left-handed patients demonstrated the same pattern of lateralized atrophy as the right-handers, suggesting that they were left-hemisphere dominant for language.

**Table 1:** Clinical and demographic features

	svPPA	nfvPPA	lvPPA	*p*
*N*	24	22	18	–
Gender/female (%)	8 (33.3)	16 (72.7)	10 (55.6)	**0.02**
Age mean ± SD, years	63.6 ± 6.7	66.1 ± 6.6	66.5 ± 6.2	0.29
Education level^a^	5.29 (0.75)	4.68 (0.95)	5.50 (1.10)	**0.01**
Handedness/right	22 (91.7)	21 (95.5)	17 (94.4)	0.86
Symptom duration mean ± SD, years	3.6 ± 1.4	2.5 ± 1.6	3.3 ± 1.5	0.06
Follow-up period mean ± SD, years	2.74 (1.49)	2.49 (1.34)	3.05 (1.23)	0.44
CDR mean ± SD	0.66 (0.28)	0.61 (0.52)	0.69 (0.25)	0.82
Reduction in ADL (%)	13 (54.2)	12 (54.5)	10 (55.6)	0.99
Genetic mutation (gene)	–	C9orf72 (*n* = 1) GRN (*n* = 1)	–	–

*svPPA* semantic variant primary progressive aphasia, *nfvPPA* non-fluent variant primary progressive aphasia, *lvPPA* logopenic variant primary progressive aphasia, *CDR* clinical dementia rating, *ADL* Activities of daily living, *GRN* progranulin, *C9orf72* chromosome 9 open-reading frame, *SD* standard deviation

^a^Verhage score

**Fig. 1:**
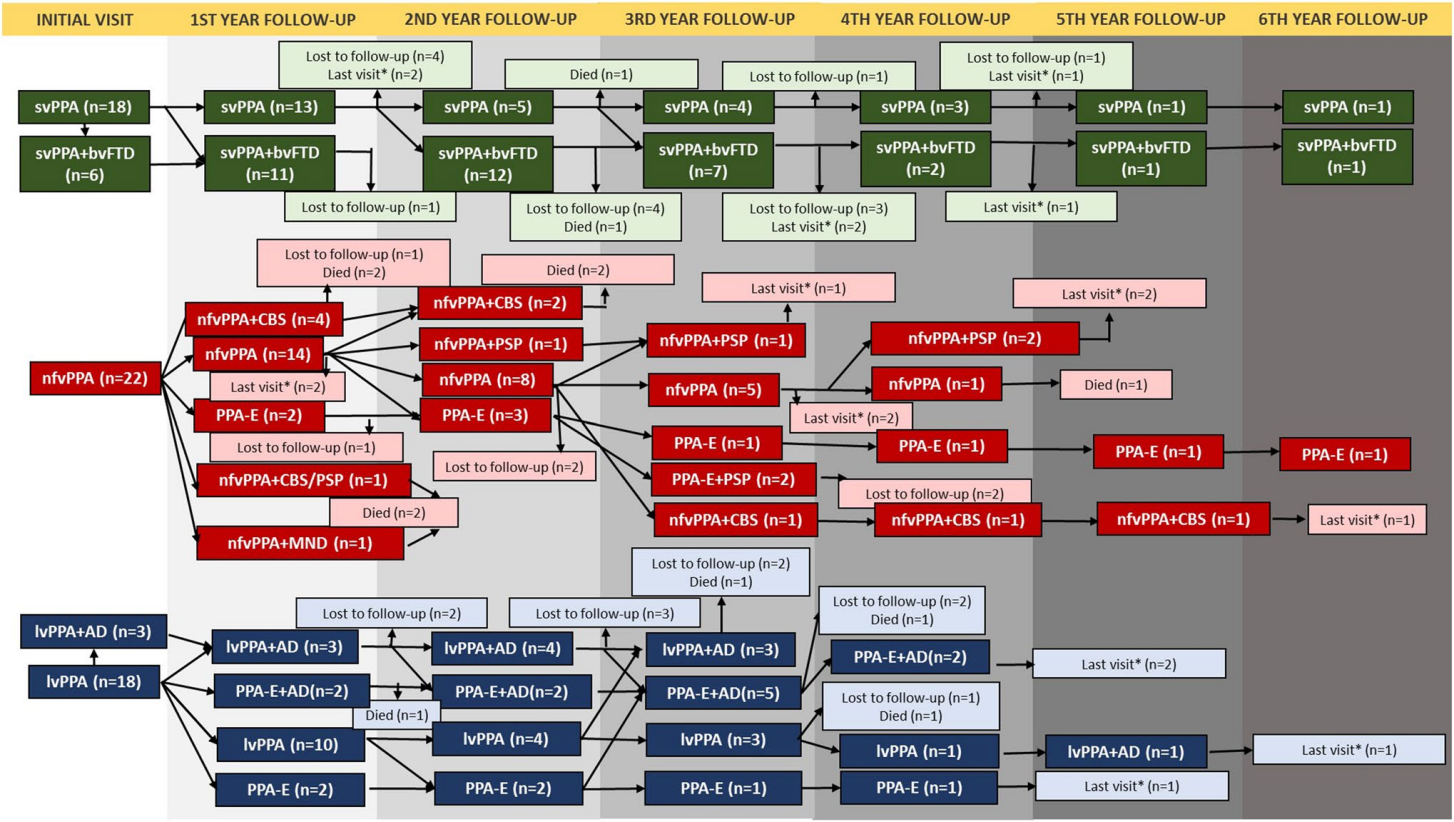
Clinical evaluation of PPA subtypes over time. *svPPA* Semantic variant primary progressive aphasia, *bvFTD* Behavioral variant frontotemporal dementia, *nfvPPA* non-fluent variant primary progressive aphasia, *PPA-E* Primary progressive aphasia-extended, *CBS* Corticobasal syndrome, *PSP* progressive supranuclear palsy, *MND* Motor neuron disease, *lvPPA* logopenic variant primary progressive aphasia, *AD* Alzheimer’s disease. *: last visit. Those subjects have been diagnosed recently and they are still under follow-up. The indicated visit is the last visit of the subject

**Fig. 2:**
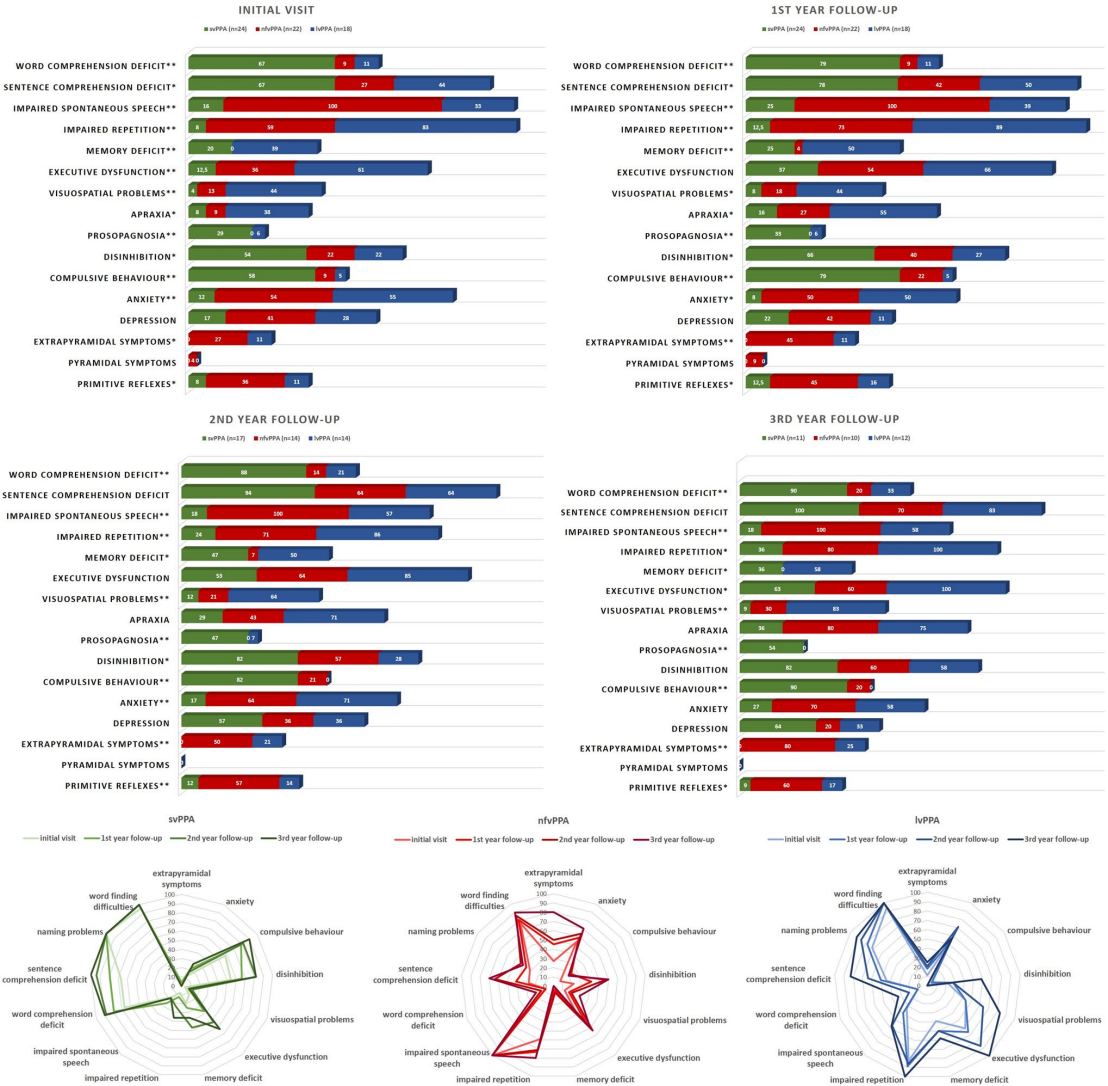
Symptom distribution of PPA subtypes over time. *svPPA* Semantic variant primary progressive aphasia, *nfvPPA* Nonfluent variant primary progressive aphasia, *lvPPA* Logopenic variant primary progressive aphasia. *p < 0.05, **p < 0.01

**Fig. 3:**
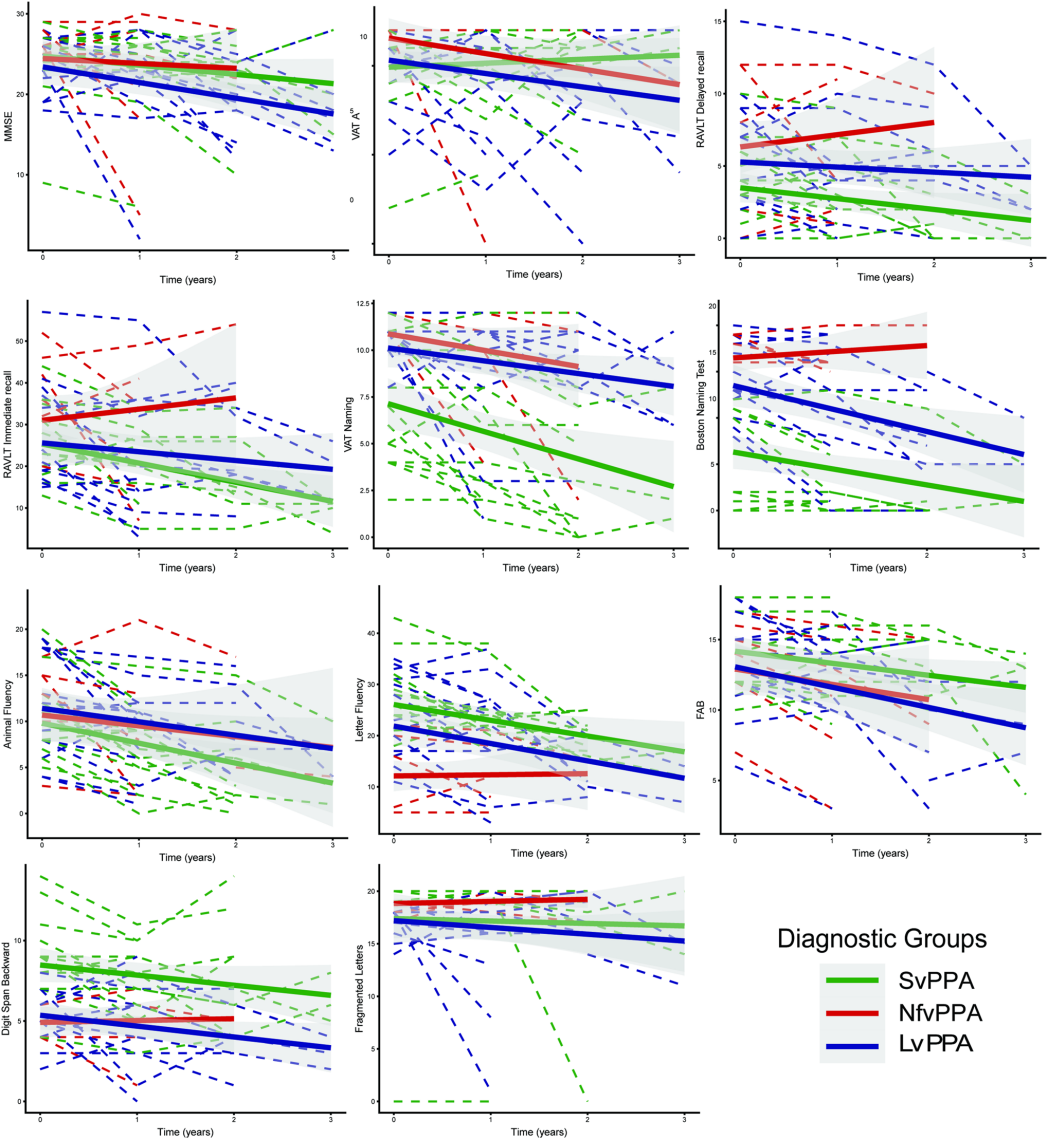
Cognitive test performance of the subtypes over time. *svPPA* Semantic variant primary progressive aphasia, *nfvPPA* Nonfluent variant primary progressive aphasia, *lvPPA* Logopenic variant primary progressive aphasia. *MMSE* mini-mental state examination, *VAT* visual association test, *RAVLT* Dutch version of the Rey Auditory Verbal Learning Test, *FAB* frontal assessment battery

A positive family history for FTD was present in one nfvPPA patient who had a hexanucleotide repeat expansion in chromosome 9 open-reading frame 72 (C9orf72) gene, and for AD in 2 lvPPA patients, whereas none of the svPPA patients had a clear autosomal dominant inheritance of any type of dementia. In none of the subjects, pathological confirmation had been achieved. Besides the patient with C9orf72 repeat expansion, another nfvPPA patient carried a pathogenic variant in the progranulin gene [c.415 T > C, p.(Cys139Arg), missense variant [29]] whose modified Goldman score was 2. Figure 1 displays the yearly clinical evaluation of each subtype with a minimum of one year, extending up to 6 years of follow-up. To ascertain the distinct clinical profile and the progression pattern of each subtype, the most pronounced symptoms are displayed in Fig. 2 and detailed longitudinal symptom distribution is displayed in supplementary material 3. Figure 3 gives an overview of the different neuropsychological test scores. Baseline scores and annual change are displayed in supplementary material 4. Additionally, initial NPI scores are displayed in supplementary Fig. 3.

### Initial clinical profiles of the three PPA variants

As expected, language difficulties were the main problem in all diagnostic groups, and since our inclusion criteria were based on the current classification system, the type of deficits were in line with the respective diagnostic criteria.

Although baseline MMSE score did not differ significantly across the subtypes, lvPPA subjects reported more widespread cognitive problems such as memory deficits (*p* < 0.01), executive dysfunction (*p* < 0.01), apraxia (*p* = 0.01), and visuospatial problems (*p* < 0.01). Moreover, they exhibited worse performance on the FAB and the VOSP fragmented letters test, indicating executive and visuospatial dysfunction, although the difference was not statistically significant. Of note, although memory deficits were reported more commonly in lvPPA, svPPA subjects performed worse on the verbal memory tests initially (Figs. 2, 3).

Behavioral problems were much more prominent in svPPA than in the other groups, especially disinhibition and compulsiveness (*p* = 0.03, *p* < 0.001 respectively). Loss of empathy and dietary changes were also more common in svPPA; however, the difference was not significant. Furthermore, loss of insight was often present in the svPPA group, whereas nvfPPA and lvPPA subjects were more aware of their symptoms (*p* = 0.001). Additionally, NPI results showed that neuropsychiatric symptoms were more prevalent in svPPA, as indicated by the scores for changing eating habits, irritability, euphoria, and disinhibition. On the other hand, nfvPPA subjects were more depressive, whereas lvPPA subjects were more anxious. However, it should be noted that regarding NPI scores, except disinhibition, the differences were not significant. Another common behavioral problem was apathy which occurred in all subtypes (Supplementary Fig. 3).

Motor symptoms were observed almost uniquely in nfvPPA. Extrapyramidal deficits were recorded in 27% of nfvPPA subjects at the initial visit, which was more common than in other groups (*p* = 0.02). One nfvPPA subject demonstrated pyramidal symptoms, whereas it was not recorded in svPPA and lvPPA.

### Progression to PPA-extended

Although linguistic problems maintained predominant in all subtypes during the disease course, patients developed various cognitive and behavioral problems as well as motor deficits. Regarding language problems, during the disease course, the nfvPPA and lvPPA patients developed several additional language problems that formally met the diagnostic criteria of another PPA syndrome, which we refer to as “PPA extended”. On the other hand, in svPPA, loss of semantic knowledge remained the main problem with a significant decline on the naming and semantic memory tests. Although the other language problems of svPPA subjects such as repetition problems and reduced spontaneous speech were not sufficient to apply PPA-extended, they showed a significant decline on the letter fluency test over time. Of note, none of the svPPA subjects progressed to mutism and dysarthria was never recorded in svPPA. Mutism was recorded in 8 subjects during follow-up of which 7 had nfvPPA. nfvPPA subjects declined on repetition as well as single word and sentence comprehension. Moreover, 4 of the nfvPPA patients also met the diagnostic criteria of svPPA (PPA-extended) over time. However, during the entire disease course, PPA-extended was the most common in lvPPA group. Over time, half of the lvPPA subjects also fulfilled the svPPA and/or nfvPPA diagnostic criteria (lvPPA + svPPA = 5, lvPPA + nfvPPA = 3, lvPPA + svPPA + nfvPPA = 1), and except one subject, all of the lvPPA-extended subjects also fulfilled the amnestic variant of the Alzheimer’s disease diagnostic criteria with underlying amyloid positivity. Additionally, nfvPPA and lvPPA patients declined significantly on the semantic memory test, however, not more than svPPA subjects.

### Progression to PPA-plus

Apart from linguistic dysfunction, global cognitive decline was observed in all groups over time, especially in svPPA and lvPPA. While svPPA and lvPPA exhibited a decline on the MMSE (*p* = 0.001), it did not decline significantly in the nfvPPA group. lvPPA subjects reported pronounced memory deficits, executive dysfunction, and visuospatial problems at the follow-up visits with a greater decline on the visual and verbal memory tests (*p* < 0.05), FAB (*p* < 0.001), digit span backward (*p* = 0.01), and VOSP fragmented letters (*p* = 0.14). However, it should be noted that svPPA subjects also showed a significant decline on the verbal memory tests and approximately half of the svPPA subjects reported episodic memory deficits (problems with remembering recent events) in the second year of the disease course. Of note, our retrospective design was not sufficient to distinguish the contribution of the semantic impairment to the episodic memory deficits. On the other hand, nfvPPA showed a relatively benign progression pattern on the cognitive tests in comparison to other subtypes; however, they developed apraxia over the disease course. Additionally, executive dysfunction became a prominent symptom for both svPPA and nfvPPA as well as lvPPA and all subtypes exhibited a significant decline on the FAB.

Even at the initial visit, behavioral changes were quite common in svPPA. Moreover, although aphasia is the most prominent symptom, 6 svPPA cases had additional behavioral problems that formally met the diagnostic criteria for possible bvFTD. During the follow-up, svPPA subjects developed even more behavioral problems. Eventually, 14 out of 24 (58%) svPPA subjects formally met the diagnostic criteria of bvFTD in their follow-up. Although both nfvPPA and lvPPA groups developed disinhibition over the disease course, compulsive behavior was observed almost uniquely in svPPA (*p* < 0.001 in all visits).

Over the disease course, motor symptoms remained more prevalent in the nfvPPA group than in the other groups. Remarkably, 80% of nfvPPA subjects had extrapyramidal signs at the third year of follow-up. However, over time, only three lvPPA subjects developed extrapyramidal symptoms, whereas it was never observed in svPPA.

From baseline to the last visit, in total, 42 patients (65.6%) additionally formally met the diagnostic criteria of another neurodegenerative syndrome, which we refer to as “PPA plus”. Median time from clinical onset to PPA-plus was 5 years (IQR = 2.5) that did not differ significantly among the subtypes. Fourteen svPPA patients met the diagnostic criteria of bvFTD (5.1 year IQR = 1.1). By contrast, nfvPPA exhibited a heterogeneous progression pattern (5.1 years IQR = 3.4). Twelve nfvPPA patients developed an atypical form of parkinsonism, of which five were categorized as progressive supranuclear palsy (PSP), six as corticobasal syndrome (CBS), and one patient with features of both PSP and CBS. In addition, two out of five subjects with PSP–PPA-plus also developed several comprehension deficits, which was referred as PPA-extended. During the entire follow-up, only one nfvPPA subject had pyramidal signs and was reclassified as MND–PPA-plus. This case, carrying a *C9orf72* mutation*,* fulfilled the diagnostic criteria of MND after 1 year of follow-up and died 3 months later. Fifteen out of 18 lvPPA patients acquired global cognitive impairment, in line with the diagnostic criteria of dementia due to Alzheimer’s disease and all of them were amyloid positive as well (4.5 years IQR = 3.4), and 8 of them were PPA-extended. In the remaining three amyloid negative lvPPA patients, language problems were more predominant and one of them developed severe comprehension deficits, and also fulfilled the diagnostic criteria of svPPA after 2 years of follow-up.

### Mortality

During the follow-up period, 12 out of 64 subjects deceased. Seven of them had nfvPPA, 3 lvPPA, and 2 svPPA.

## Discussion

In this retrospective longitudinal cohort study to compare the natural history between PPA subtypes, we investigated overlapping and distinguishing clinical features, and progression pattern of the three PPA subtypes. Even though aphasia is by definition the earliest and common feature of the syndrome, our results highlighted that PPA constitutes a heterogeneous clinical syndrome and additional cognitive, behavioral, and motor deficits emerge with time. After diagnosis, each subtype exhibited a typical progression pattern (PPA-extended and PPA-plus). Whereas a strong relationship existed between svPPA and the clinical features of bvFTD, subjects with nfvPPA developed motor impairment and progressed into various forms of neurodegenerative syndromes such as CBS, PSP, and MND. Patients with lvPPA progressed with multiple cognitive domain deficits into Alzheimer’s disease dementia at follow-up, and PPA-extended forms were more common in lvPPA, especially in the amyloid positive group.

Regarding linguistic problems, over time, nfvPPA and lvPPA patients developed symptoms that exceeded the core criteria, whereas svPPA patients did not. At a closer look, in accordance with the previous longitudinal studies, lvPPA declined on repetition [30–32], naming [30–32], comprehension [32], and speech production [14, 31], while nfvPPA declined on comprehension and repetition [13]. On the other hand, the most important change over time in svPPA was the development of sentence comprehension problems, which has been reported previously [30, 33]. Although svPPA patients declined on the letter fluency test just like was reported in a recent longitudinal study [14], they were more fluent compare to the other subtypes and neither mutism/dysarthria nor PPA-extended was observed in svPPA.

Although not mentioned in the diagnostic criteria of svPPA [2], it is common knowledge that behavioral changes similar to those occurring in bvFTD are often evident at presentation in these patients [11, 34–36]. Consistent with this observation, particularly, disinhibition and compulsive behavior were common in svPPA, next to irritability, euphoria, and a change in eating habits. Supporting the association between compulsive behavior and temporal lobes [20, 21, 37], compulsiveness was observed uniquely in svPPA both initial and follow-up visits. Additionally, in line with earlier studies [34, 38], apathy was common in all subtypes and lvPPA and nfPPA subjects were more aware of their symptoms, which might be related to feeling more anxiety, in contrast to svPPA [35, 38, 39].

Remarkably, in comparison with the other PPA subtypes, lvPPA displayed a broad range of initial cognitive problems such as memory deficits, apraxia, executive, and visuospatial dysfunction and a more rapid and generalized cognitive decline over the disease course which was also confirmed in the smaller subgroup that received the longitudinal cognitive tests, consistent with previous work [40–43]. However, executive dysfunction became one of the prominent symptoms in svPPA and nfvPPA as well as lvPPA over the disease course. In addition, svPPA demonstrated verbal memory impairment, whereas nfvPPA developed apraxia over time.

One of the important results of our study is that despite the relatively benign early presentation, a high proportion of nfvPPA cases developed motor disturbances such as MND, PSP, and CBS and it is conceivable that these syndromes increase mortality risk. Although, only 12 patients deceased in the follow-up and a larger sample size longitudinal study has reported a longer survival in nfvPPA [44], and a large body of literature has showed a significant shorter survival in FTD patients with motor disturbances [45, 46]. The relationship between pyramidal, extrapyramidal symptoms, and nfvPPA have been reported previously [11, 47–50], and some authors have suggested that apraxia of speech is the clinical marker of progression to PSP and CBS [48]. Although apraxia of speech was not evaluated in individual patients in this retrospective study, in line with the literature [51], mutism was recorded much more often in nfvPPA than in other subtypes.

Compared with other cohort studies, our sample was older than an American population-based cohort [52], however younger than other European population-based patient groups [14, 17, 18]. Our svPPA sample was male predominant, whereas the nfvPPA sample was female predominant. Although the general assumption is that PPA occurs with approximately equal prevalence across sexes [9], sex distribution has shown a variety in the previous studies and there is no solid consistency [17, 18, 52].

This is the first biomarker-based, systematic longitudinal cohort study from a well-structured dementia clinic that provides detailed symptomatology, and progression pattern of each PPA subtype. However, there are some limitations that should be addressed. First of all, the study was performed retrospectively, and since we adhere to the most recent diagnostic criteria of 2011, our sample size is relatively small. Secondly, longitudinal NPI data were not available, and because of drop-outs and progression, the eligible longitudinal cognitive test data were limited. A larger sample size would be helpful to evaluate the underlying risk factors and clinical predictors of progression to PPA-plus and mortality. Another limitation might be the lack of genetic or pathological confirmation. However, we provided the amyloid status of each patient that is informative about underlying Alzheimer’s disease pathology. Moreover, showing the progression pattern of FTD related genetic and/or pathological subtypes is beyond the scope of this study. The main aim of the study is giving an overview to clinicians about the progression pattern of the well-identified PPA subtypes based on the current clinical diagnostic criteria. For this purpose, we used the terms PPA-extended and PPA-plus to emphasize that those patients had a primary PPA diagnosis initially and developed additional symptoms. Emphasizing the evolution of new symptoms that lead to a secondary, parallel diagnosis might facilitate the recognition of the various PPA subtypes. Additionally, our results support the recent argument, suggesting that FTLD syndromes are not discrete in the clinical features of their respective clinical criteria, but instead exist as a multidimensional spectrum [53]. Note that this is not an argument for creating new labeling systems or new subtypes; however, it might be a useful answer of one important question; what should we expect next?

In conclusion, although, by definition, aphasia is the only and predominant symptom in PPA [1, 2], it does not take long before other symptoms occur. More importantly, its progression pattern is subtype-specific. Although svPPA seems to be more homogeneous with respect to its language profile, healthcare providers and caregivers should be aware of behavioral disturbances that might arise, whereas global cognitive decline and broad language problems due to underlying Alzheimer’s disease pathology should be expected for lvPPA. nfvPPA patients may be least affected on the behavioral and cognitive domains initially, but show a progression to other neurodegenerative syndromes, particularly those associated with motor impairment which might cause a high mortality risk.

## Supplementary Information

Below is the link to the electronic supplementary material.

Supplementary file1 Supplementary Figure 1: Atrophy pattern of the subtypes. svPPA; Semantic variant primary progressive aphasia. Predominant left anterior temporal atrophy. *nfvPPA* Nonfluent variant primary progressive aphasia. Predominant left posterior fronto-insular atrophy. *lvPPA* Logopenic variant primary progressive aphasia. Predominant left posterior perisylvian atrophy, *L* Left (TIF 2874 KB)

Supplementary file2 Supplementary Figure 2: Patient selection scheme. *PPA* Primary progressive aphasia, *rtvFTD* Right temporal variant frontotemporal dementia, *svPPA* Semantic variant primary progressive aphasia, *nfvPPA* Nonfluent variant primary progressive aphasia, *lvPPA* Logopenic variant primary progressive aphasia (JPG 110 KB)

Supplementary file3 Supplementary Figure 3: Neuropsychiatric inventory scores of the subtypes. *svPPA* Semantic variant primary progressive aphasia, *nfvPPA* Nonfluent variant primary progressive aphasia, *lvPPA* Logopenic variant primary progressive aphasia, *NBD* Night-time behavioral disturbances, *AMB* Aberrant motor behavior (TIF 4749 KB)

Supplementary file4 (DOCX 17 KB)

Supplementary file5 (DOCX 12 KB)

Supplementary file6 (DOCX 16 KB)

Supplementary file7 (DOCX 18 KB)

## Data Availability

The corresponding author has full access to all data and materials and can provide availability if needed.

## Code availability

SPSS version 24.0 (IBM) and R Studio (R Core Team, 2018).
