# Supplementary figures and images for: The natural history of primary progressive aphasia: beyond aphasia
Source: J Neurol. 2021 Jul 3;269(3):1375–85. doi: 10.1007/s00415-021-10689-1 (PMC8857134; doi:10.1007/s00415-021-10689-1)

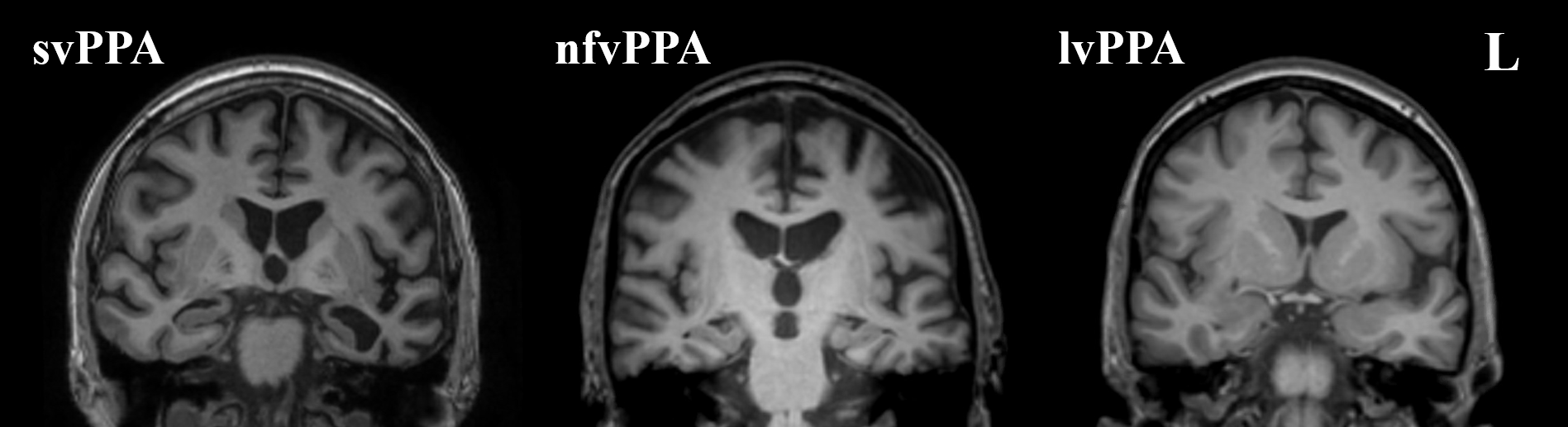

Supplement: Supplementary file 1 — Supplementary file1 Supplementary Figure 1: Atrophy pattern of the subtypes. svPPA; Semantic variant primary progressive aphasia. Predominant left anterior temporal atrophy. nfvPPA Nonfluent variant primary progressive aphasia. Predominant left posterior fronto-insular atrophy. lvPPA Logopenic variant primary progressive aphasia. Predominant left posterior perisylvian atrophy, L Left (TIF 2874 KB) [file 415_2021_10689_MOESM1_ESM.tif]

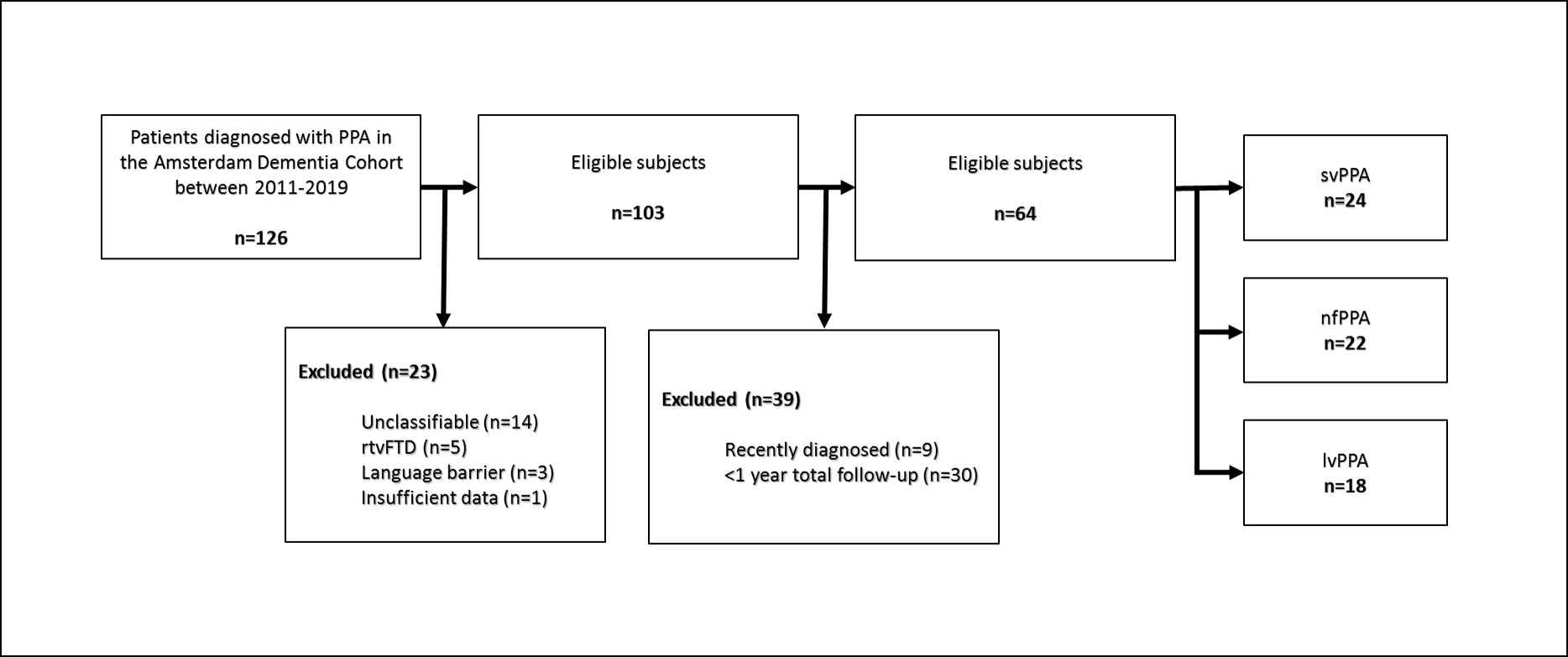

Supplement: Supplementary file 2 — Supplementary file2 Supplementary Figure 2: Patient selection scheme. PPA Primary progressive aphasia, rtvFTD Right temporal variant frontotemporal dementia, svPPA Semantic variant primary progressive aphasia, nfvPPA Nonfluent variant primary progressive aphasia, lvPPA Logopenic variant primary progressive aphasia (JPG 110 KB) [file 415_2021_10689_MOESM2_ESM.jpg]

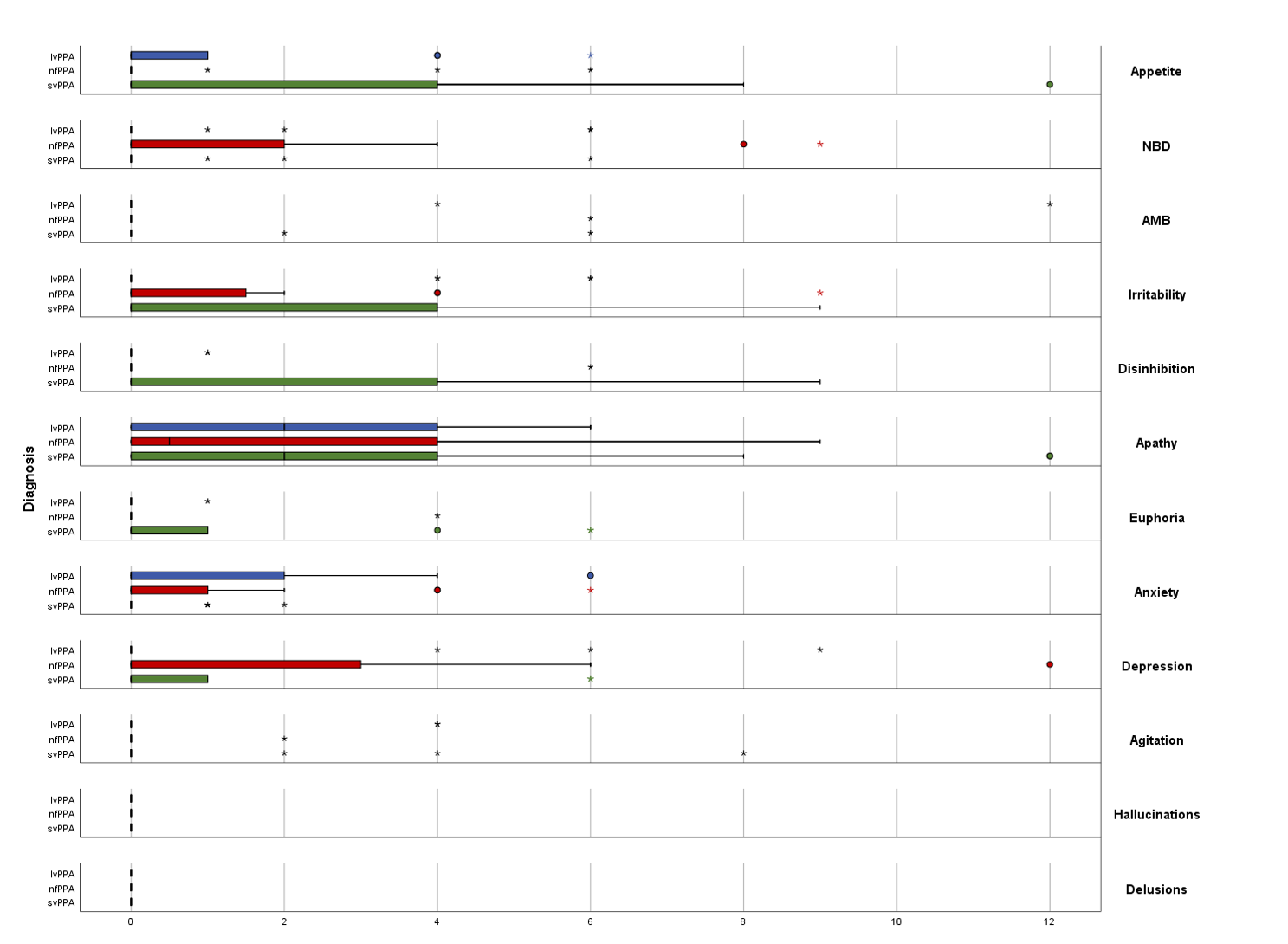

Supplement: Supplementary file 3 — Supplementary file3 Supplementary Figure 3: Neuropsychiatric inventory scores of the subtypes. svPPA Semantic variant primary progressive aphasia, nfvPPA Nonfluent variant primary progressive aphasia, lvPPA Logopenic variant primary progressive aphasia, NBD Night-time behavioral disturbances, AMB Aberrant motor behavior (TIF 4749 KB) [file 415_2021_10689_MOESM3_ESM.tif]
