# Supplementary material for: The natural history of primary progressive aphasia: beyond aphasia
Source: J Neurol. 2021 Jul 3;269(3):1375–85. doi: 10.1007/s00415-021-10689-1 (PMC8857134; doi:10.1007/s00415-021-10689-1)
Supplement: Supplementary file 4 — Supplementary file4 (DOCX 17 KB) [file 415_2021_10689_MOESM4_ESM.docx]

**Supplementary Material 1: Amyloid status of the subjects**

| **Diagnosis** | **CSF_AB42 (**pg/mL) | **CSF_Tau (**pg/mL) | **CSF_p-Tau (**pg/mL) | **PET scan** | **PET Amyloid** | **Amyloid status** |
| --- | --- | --- | --- | --- | --- | --- |
| svPPA | 632 (H) | 107 (L) | n.a. | n.a. | n.a. | Non-indicative of AD |
| svPPA | 895 (H) | 331 (L) | 39 (L) | n.a. | n.a. | Non-indicative of AD |
| svPPA | 1047 (H) | 247 (L) | 40 (L) | n.a. | n.a. | Non-indicative of AD |
| svPPA | 1098 (H) | 358 (L) | 44 (L) | n.a. | n.a. | Non-indicative of AD |
| svPPA | n.a. | n.a. | n.a. | Flutemetamol | Negative | Non-indicative of AD |
| svPPA | 1098 (H) | 413 (H) | 45 (L) | n.a. | n.a. | Non-indicative of AD |
| svPPA | n.a. | n.a. | n.a. | Flutemetamol | Negative | Non-indicative of AD |
| svPPA | n.a. | n.a. | n.a. | Flutemetamol | Negative | Non-indicative of AD |
| svPPA | 561 (H) | 621 (H) | 65 (H) | n.a. | n.a. | Non-indicative of AD |
| svPPA | 523 (L) | 272 (L) | 51 (L) | PIB | Negative | Non-indicative of AD |
| svPPA | 929 (H) | 154 (L) | 24 (L) | n.a. | n.a. | Non-indicative of AD |
| svPPA | 1351 (H) | 600 (H) | 66 (H) | n.a. | n.a. | Non-indicative of AD |
| svPPA | 341 (L) | 188 (L) | 181 (H) | Flutemetamol | Negative | Non-indicative of AD |
| svPPA | 1239 (H) | 409 (H) | 47 (L) | n.a. | n.a. | Non-indicative of AD |
| svPPA | 1012 (H) | 289 (L) | 39 (L) | Florbetaben | Negative | Non-indicative of AD |
| svPPA | 535 (L) | 593 (H) | 60 (H) | Florbetaben | Negative | Non-indicative of AD |
| svPPA | 759 (H) | 630 (H) | 39 (L) | Florbetaben | Negative | Non-indicative of AD |
| svPPA | 816 (H) | 557 (H) | 54 (H) | n.a. | n.a. | Non-indicative of AD |
| svPPA | 1538 (H) | 466 (H) | 50 (L) | n.a. | n.a. | Non-indicative of AD |
| svPPA | n.a. | n.a. | n.a. | PIB | Negative | Non-indicative of AD |
| svPPA | 1235 (H) | 403 (H) | 58 (H) | n.a. | n.a. | Non-indicative of AD |
| svPPA | n.a. | n.a. | n.a. | PIB | Negative | Non-indicative of AD |
| svPPA | n.a. | n.a. | n.a. | Florbetaben | Negative | Non-indicative of AD |
| svPPA | 1210 (H) | 254 (L) | 42 (L) | n.a. | n.a. | Non-indicative of AD |
| nfvPPA | 1075 (H) | 301 (L) | 43 (L) | n.a. | n.a. | Non-indicative of AD |
| nfvPPA | n.a. | n.a. | n.a. | PIB | Negative | Non-indicative of AD |
| nfvPPA | n.a. | n.a. | n.a. | PIB | Negative | Non-indicative of AD |
| nfvPPA | 1614 (H) | 476 (H) | 41 (L) | n.a. | n.a. | Non-indicative of AD |
| nfvPPA | 843 (H) | 317 (L) | 35 (L) | n.a. | n.a. | Non-indicative of AD |
| nfvPPA | 1653 (H) | 844 (H) | 50 (H) | n.a. | n.a. | Non-indicative of AD |
| nfvPPA | 487 (L) | 559 (H) | 56 (H) | Flutemetamol | Positive | Indicative of AD |
| nfvPPA | 534 (L) | 356 (L) | 44 (L) | PIB | Negative | Non-indicative of AD |
| nfvPPA | 497 (L) | 140 (L) | 27 (L) | PIB | Negative | Non-indicative of AD |
| nfvPPA | n.a. | n.a. | n.a. | PIB | Negative | Non-indicative of AD |
| nfvPPA | 1013 (H) | 278 (L) | 34 (L) | n.a. | n.a. | Non-indicative of AD |
| nfvPPA | 699 (H) | 459 (H) | 50 (L) | n.a. | n.a. | Non-indicative of AD |
| nfvPPA | 1160 (H) | 621 (H) | 69 (H) | n.a. | n.a. | Non-indicative of AD |
| nfvPPA | 1057 (H) | 247 (L) | 34 (L) | Flutemetamol | Negative | Non-indicative of AD |
| nfvPPA | 850 (H) | 225 (L) | 40 (L) | n.a. | n.a. | Non-indicative of AD |
| nfvPPA | 872 (H) | 267 (L) | 34 (L) | n.a. | n.a. | Non-indicative of AD |
| nfvPPA | 1272 (H) | 237 (L) | 40 (L) | n.a. | n.a. | Non-indicative of AD |
| nfvPPA | 1358 (H) | 526 (H) | 73 (H) | n.a. | n.a. | Non-indicative of AD |
| nfvPPA | 1078 (H) | 306 (L) | 41 (L) | n.a. | n.a. | Non-indicative of AD |
| nfvPPA | 764 (H) | 261 (L) | 24 (L) | n.a. | n.a. | Non-indicative of AD |
| nfvPPA | 1144 (H) | 333 (L) | 54 (H) | n.a. | n.a. | Non-indicative of AD |
| nfvPPA | 1700 (H) | 288 (L) | 43 (L) | n.a. | n.a. | Non-indicative of AD |
| lvPPA | 397 (L) | 275 (L) | 53 (H) | PIB | Positive | Indicative of AD |
| lvPPA | 445 (L) | 877 (H) | 81 (H) | PIB | Positive | Indicative of AD |
| lvPPA | 566 (H) | 496 (H) | 62 (H) | PIB | Positive | Indicative of AD |
| lvPPA | 428 (L) | 921 (H) | 99 (H) | Flutemetamol | Positive | Indicative of AD |
| lvPPA | 553 (H) | 627 (H) | 78 (H) | PIB | Positive | Indicative of AD |
| lvPPA | 305 (L) | 962 (H) | 117 (H) | Flutemetamol | Positive | Indicative of AD |
| lvPPA | 555 (H) | 402 (H) | 52 (H) | PIB | Positive | Indicative of AD |
| lvPPA | 538 (L) | 356 (L) | 46 (L) | Flutemetamol | Positive | Indicative of AD |
| lvPPA | 561 (H) | 479 (H) | 65 (H) | Flutemetamol | Positive | Indicative of AD |
| lvPPA | 621 (H) | 369 (L) | 53 (H) | Flutemetamol | Positive | Indicative of AD |
| lvPPA | 647 (H) | 1510 (H) | 133 (H) | n.a. | n.a. | Non-indicative of AD |
| lvPPA | n.a. | n.a. | n.a. | PIB | Positive | Indicative of AD |
| lvPPA | 926 (H) | 407 (H) | 78 (H) | n.a. | n.a. | Non-indicative of AD |
| lvPPA | 528 (L) | 720 (H) | 106 (H) | n.a. | n.a. | Indicative of AD |
| lvPPA | 514 (L) | 1154 (H) | 90 (H) | Flutemetamol | Positive | Indicative of AD |
| lvPPA | 445 (L) | 592 (H) | 82 (H) | Florbetaben | Positive | Indicative of AD |
| lvPPA | 620 (H) | 590 (H) | 83 (H) | n.a. | n.a. | Non-indicative of AD |
| lvPPA | 619 (H) | 827 (H) | 105 (H) | Flutemetamol | Positive | Indicative of AD |

svPPA: semantic variant primary progressive aphasia, nfvPPA: non-fluent variant primary progressive aphasia, lvPPA: logopenic variant primary progressive aphasia, AD: Alzheimer’s disease, CSF: cerebrospinal fluid, PET: Positron emission tomography, PIB: Pittsburgh compound B, H: High, L: Low, n.a.: not available
