# Supplementary material for: The natural history of primary progressive aphasia: beyond aphasia
Source: J Neurol. 2021 Jul 3;269(3):1375–85. doi: 10.1007/s00415-021-10689-1 (PMC8857134; doi:10.1007/s00415-021-10689-1)
Supplement: Supplementary file 5 — Supplementary file5 (DOCX 12 KB) [file 415_2021_10689_MOESM5_ESM.docx]

**Supplementary material 2: Categorization of symptoms**

| **Language/speech** | **Cognitive** | **Behaviour/ mood** | **Motor** |
| --- | --- | --- | --- |
| Word finding difficulties | Memory deficit | Disinhibition | Pyramidal signs |
| Sentence comprehension deficit | Prosopagnosia | Loss of insight | Extrapyramidal signs |
| Single word comprehension deficit | Executive dysfunction | Compulsive behaviour | Primitive reflexes |
| Dyslexia/dysgraphia | Apraxia | Apathy/ inertia | Swallowing problems |
| Spontaneous speech impairment | Visuospatial problems | Hyper-orality and changing eating habits | Falling |
| Naming problems |  | Loss of empathy | Eye movement impairment |
| Impaired repetition |  | Depression |  |
| Impaired object knowledge |  | Anxiety |  |
| Dysarthria |  |  |  |
| Mutism |  |  |  |
