# Supplementary material for: The natural history of primary progressive aphasia: beyond aphasia
Source: J Neurol. 2021 Jul 3;269(3):1375–85. doi: 10.1007/s00415-021-10689-1 (PMC8857134; doi:10.1007/s00415-021-10689-1)
Supplement: Supplementary file 6 — Supplementary file6 (DOCX 16 KB) [file 415_2021_10689_MOESM6_ESM.docx]

**Supplementary material 3: Clinical features of the diagnostic groups**

| **Symptoms** | **Initial visit (%)** | | | **1^st^ year follow-up (%)** | | | **2^nd^ year follow-up (%)** | | | **3^rd^ year follow-up (%)** | | |
| --- | --- | --- | --- | --- | --- | --- | --- | --- | --- | --- | --- | --- |
| **Language/ Speech** | **svPPA**  **(n=24)** | **nfPPA**  **(n=22)** | **lvPPA**  **(n=18)** | **svPPA**  **(n=24)** | **nfPPA**  **(n=22)** | **lvPPA**  **(n=18)** | **svPPA**  **(n=17)** | **nfPPA**  **(n=14)** | **lvPPA**  **(n=14)** | **svPPA**  **(n=11)** | **nfPPA**  **(n=10)** | **lvPPA**  **(n=12)** |
| Word finding difficulties | 96 | 77 | 94 | 100 | 81 | 100 | 100 | 86 | 100 | 100 | 90 | 100 |
| Sentence comprehension deficit | 67 | 27 | 44 | 79 | 41 | 50 | 94 | 64 | 64 | 100 | 70 | 83 |
| Single word comprehension deficit | 67 | 9 | 11 | 79 | 9 | 11 | 88 | 14 | 21 | 90 | 20 | 33 |
| Dyslexia/dysgraphia | 37 | 50 | 50 | 46 | 63 | 72 | 65 | 86 | 93 | 63 | 90 | 100 |
| Spontaneous speech impairment | 16 | 100 | 33 | 25 | 100 | 39 | 18 | 100 | 57 | 18 | 100 | 58 |
| Naming problems | 100 | 31 | 72 | 100 | 36 | 78 | 100 | 43 | 86 | 100 | 40 | 92 |
| Impaired repetition | 8 | 59 | 83 | 12 | 73 | 89 | 24 | 71 | 86 | 36 | 80 | 100 |
| Impaired object knowledge | 67 | 0 | 6 | 75 | 0 | 6 | 71 | 0 | 7 | 54 | 0 | 8 |
| Dysarthria | 0 | 41 | 6 | 0 | 46 | 6 | 0 | 36 | 7 | 0 | 40 | 8 |
| Mutism | 0 | 0 | 0 | 0 | 4 | 0 | 0 | 0 | 0 | 0 | 40 | 0 |
| **Cognitive** |  |  |  |  |  |  |  |  |  |  |  |  |
| Memory deficit | 20 | 0 | 39 | 25 | 4 | 50 | 47 | 7 | 50 | 36 | 0 | 58 |
| Prosopagnosia | 29 | 0 | 6 | 33 | 0 | 6 | 47 | 0 | 7 | 54 | 0 | 0 |
| Executive dysfunction | 12 | 36 | 61 | 37 | 54 | 66 | 53 | 64 | 86 | 63 | 60 | 100 |
| Apraxia | 8 | 9 | 38 | 17 | 27 | 56 | 29 | 43 | 71 | 36 | 80 | 75 |
| Visuospatial problems | 4 | 13 | 44 | 8 | 18 | 44 | 12 | 21 | 64 | 9 | 30 | 83 |
| **Behavioural/ mood** |  |  |  |  |  |  |  |  |  |  |  |  |
| Disinhibition | 54 | 22 | 22 | 66 | 41 | 28 | 82 | 57 | 28 | 82 | 60 | 58 |
| Loss of insight | 46 | 0 | 17 | 58 | 9 | 28 | 65 | 14 | 36 | 64 | 20 | 42 |
| Compulsive behaviour | 58 | 9 | 5 | 79 | 23 | 6 | 82 | 21 | 0 | 90 | 20 | 0 |
| Apathy/ inertia | 58 | 50 | 50 | 66 | 63 | 55 | 65 | 71 | 50 | 63 | 80 | 41 |
| Hyper-orality and changing eating habits | 37 | 13 | 28 | 37 | 18 | 28 | 41 | 21 | 21 | 45 | 20 | 17 |
| Loss of empathy | 25 | 9 | 6 | 33 | 14 | 6 | 47 | 21 | 7 | 45 | 20 | 8 |
| Depression | 17 | 41 | 28 | 21 | 45 | 28 | 47 | 36 | 36 | 64 | 20 | 33 |
| Anxiety | 12 | 54 | 55 | 21 | 59 | 55 | 17 | 64 | 71 | 27 | 70 | 58 |
| **Motor** |  |  |  |  |  |  |  |  |  |  |  |  |
| Pyramidal signs | 0 | 4 | 0 | 0 | 4 | 0 | 0 | 0 | 0 | 0 | 0 | 0 |
| Extrapyramidal signs | 0 | 27 | 11 | 4 | 45 | 18 | 0 | 50 | 21 | 0 | 80 | 25 |
| Primitive reflexes | 8 | 36 | 11 | 12 | 45 | 16 | 12 | 57 | 14 | 9 | 60 | 17 |
| Swallowing problems | 0 | 18 | 0 | 0 | 23 | 0 | 0 | 7 | 0 | 0 | 0 | 0 |
| Falling | 0 | 4 | 0 | 0 | 14 | 0 | 0 | 29 | 0 | 0 | 20 | 8 |
| Eye movement impairment | 0 | 9 | 0 | 0 | 23 | 0 | 0 | 29 | 0 | 0 | 40 | 0 |
