# Supplementary material for: The natural history of primary progressive aphasia: beyond aphasia
Source: J Neurol. 2021 Jul 3;269(3):1375–85. doi: 10.1007/s00415-021-10689-1 (PMC8857134; doi:10.1007/s00415-021-10689-1)
Supplement: Supplementary file 7 — Supplementary file7 (DOCX 18 KB) [file 415_2021_10689_MOESM7_ESM.docx]

**Supplementary Material 4**

**Table 1: Baseline cognitive test scores of the diagnostic groups**

|  |  | | **svPPA** | | **nfvPPA** | | **lvPPA** | | **One-way ANOVA** | |
| --- | --- | --- | --- | --- | --- | --- | --- | --- | --- | --- |
|  |  | | **N** | **Mean±SD** | **N** | **Mean±SD** | **N** | **Mean±SD** | **P** | **Group differences** |
|  | **Global cognition** | MMSE | 24 | 24.83±4.29 | 22 | 24.68±4.21 | 18 | 23.44±3.43 | 0.503 | NS |
| **Episodic memory** | **Visual memory** | VAT A | 22 | 10.14±2.57 | 20 | 11.50±1.57 | 18 | 10.22±2.16 | 0.089 | NS |
|  | **Verbal memory** | RAVLT-Immediate recall | 21 | 24.67±9.36 | 19 | 31.95±11.51 | 18 | 26.22±12.74 | 0.111 | NS |
|  |  | RAVLT-Delayed recall | 21 | 3.24±2.47 | 19 | 6.42±3.70 | 18 | 5.44±4.09 | **0.015** | nfvPPA, lvPPA>svPPA |
| **Language** | **Naming** | VAT naming | 23 | 7.26±3.48 | 20 | 10.80±2.12 | 18 | 10.50±1.38 | **<0.001** | nfvPPA, lvPPA>svPPA |
|  |  | Boston naming | 24 | 6.46±2.47 | 18 | 16.50±2.56 | 18 | 13.38±2.38 | **<0.001** | nfvPPA, lvPPA>svPPA |
|  | **Semantic memory** | Animal fluency | 23 | 10.04±4.87 | 19 | 10.63±6.44 | 18 | 11.67±5.35 | 0.649 | NS |
|  | **Fluency** | Letter fluency | 23 | 25.70±9.83 | 17 | 12.06±6.19 | 18 | 20.39±9.38 | **<0.001** | svPPA, lvPPA>nfvPPA |
| **Executive function** | **Global executive function** | FAB | 23 | 14.13±2.46 | 20 | 12.95±4.30 | 16 | 12.81±3.60 | 0.412 | NS |
|  | **Working memory** | Digit span backward | 23 | 8.52±2.97 | 18 | 4.83±2.01 | 17 | 5.35±1.77 | **<0.001** | svPPA > lvPPA, nfvPPA |
| **Visuospatial function** | **Visuospatial function** | VOSP- Fragmented letters | 19 | 17.59±1.49 | 18 | 18.78±0.81 | 16 | 17.44±1.71 | 0.563 | NS |

svPPA; semantic variant primary progressive aphasa, nfvPPA; non fluent variant primary progressive aphasia, lvPPA; logopenic variant primary progressive aphasia, MMSE; mini mental state examination, VAT; visual association test, RAVLT; Dutch version of the Rey Auditory Verbal Learning Test, FAB; frontal assessment battery, VOSP; Visual objective and space perception

**Table 2: Annual change over time (Linear Mixed Model)**

|  |  |  | **svPPA** | | | **nfvPPA** | | | **lvPPA** | | |
| --- | --- | --- | --- | --- | --- | --- | --- | --- | --- | --- | --- |
|  |  |  | **N** | **Estimates (95% CI)** | **P** | **N** | **Estimates (95% CI)** | **P** | **N** | **Estimates (95% CI)** | **P** |
|  | **Global cognition** | MMSE | 24 | -1.46 (-2.23 – -0.68 ) | **0.001** | 22 | -0.84 (-3.60 – 1.92) | 0.556 | 18 | -2.25 (-3.48 – -1.01) | **0.001** |
| **Episodic memory** | **Visual memory** | VATA | 22 | -0.04 (-0.56 – 0.48) | 0.883 | 20 | -0.92 (-1.90 – 0.05) | 0.073 | 18 | -0.93 (-1.55 – -0.30) | **0.006** |
|  | **Verbal memory** | RAVLT-Immediate recall | 21 | -4.24 (-6.09 – -2.39) | **<0.001** | 19 | -0.76 (-9.35 – 7.82) | 0.864 | 18 | -4.04 (-6.42 – -1.65) | **0.002** |
|  |  | RAVLT-Delayed recall | 21 | -0.90 (-1.46 – -0.35) | **0.003** | 19 | -0.46 (-2.46 – 1.53) | 0.657 | 18 | -1.08 (-1.75 – -0.41) | **0.004** |
| **Language** | **Naming** | VAT naming | 23 | -1.59 (-2.18 – -1.01) | **<0.001** | 20 | -1.06 (-2.30 – 0.17) | 0.106 | 18 | -0.85 (-1.47 – -0.24) | **0.010** |
|  |  | Boston naming | 24 | -1.73 (-2.61 – -0.85) | **0.001** | 18 | -0.63 (-1.69 – 0.44) | 0.292 | 18 | -2.68 (-3.66 – -1.71) | **<0.001** |
|  | **Semantic memory** | Animal fluency | 23 | -2.47 (-3.21 – -1.74) | **<0.001** | 19 | -2.86 (-5.08 – -0.63) | **0.030** | 18 | -2.03 (-3.07 – -0.99) | **0.001** |
|  | **Fluency** | Letter fluency | 23 | -3.76 (-5.25 – -2.27) | **<0.001** | 17 | -2.19 (-4.70 – 0.31) | 0.125 | 18 | -4.19 (-5.94 – -2.44) | **<0.001** |
| **Executive function** | **Global executive function** | FAB | 23 | -0.89 (-1.58 – -0.20) | **0.015** | 20 | -2.31 (-3.12 – -1.51) | **<0.001** | 16 | -1.85 (-2.62 – -1.09) | **<0.001** |
|  | **Working memory** | Digit span backward | 23 | -0.61 (-1.14 – -0.09) | **0.029** | 18 | -0.51 (-1.36 – 0.34) | 0.263 | 17 | -0.68 (-1.19 – -0.17) | **0.013** |
| **Visuospatial function** | **Visuospatial function** | VOSP- Fragmented letters | 19 | -0.97 (-2.39 – 0.44) | 0.191 | 18 | 0.14 (-0.30 – 0.57) | 0.543 | 16 | -0.88 (-2.03 – 0.27) | 0.143 |

svPPA; semantic variant primary progressive aphasa, nfvPPA; non fluent variant primary progressive aphasia, lvPPA; logopenic variant primary progressive aphasia, MMSE; mini mental state examination, VAT; visual association test, RAVLT; Dutch version of the Rey Auditory Verbal Learning Test, FAB; frontal assessment battery, VOSP; Visual objective and space perception
